# T cell response specificity and magnitude against SIVmac239 are not concordant in major histocompatibility complex-matched animals

**DOI:** 10.1186/1742-4690-10-116

**Published:** 2013-10-24

**Authors:** Brian T Cain, Ngoc H Pham, Melisa L Budde, Justin M Greene, Jason T Weinfurter, Matthew Scarlotta, Max Harris, Emily Chin, Shelby L O’Connor, Thomas C Friedrich, David H O’Connor

**Affiliations:** 1 Department of Pathology and Laboratory Medicine, University of Wisconsin, Madison, Wisconsin, USA; 2 Wisconsin National Primate Research Center, Madison, Wisconsin, USA; 3 Department of Pathobiological Sciences, University of Wisconsin, Madison, Wisconsin, USA; 4 Department of Cellular and Molecular Biology, University of Wisconsin, Madison, Wisconsin, USA

**Keywords:** SIVmac239, MCM, ELISPOT, T cell, Reproducibility

## Abstract

**Background:**

CD8+ T cell responses, restricted by major histocompatibility complex (MHC) class I molecules, are critical to controlling human immunodeficiency virus type 1 (HIV-1) and simian immunodeficiency virus (SIV) replication. Previous studies have used MHC-matched siblings and monozygotic twins to evaluate genetic and stochastic influences on HIV-specific T cell responses and viral evolution. Here we used a genetically restricted population of Mauritian cynomolgus macaques (MCM) to characterize T cell responses within nine pairs of MHC-matched animals.

**Findings:**

In MHC-matched animals, there was considerable heterogeneity in the specificity and magnitude of T cell responses detected via individual peptide gamma interferon (IFN-γ) enzyme-linked immunospot (ELISPOT) assays. These findings were further supported by full proteome pooled peptide matrix ELISPOT data collected from this cohort at 52 weeks post-infection. Interestingly, peptide regions that elicited dominant T cell responses were more commonly shared between MHC-matched MCM than peptide regions that elicited non-dominant T cell responses.

**Conclusions:**

Our findings suggest that, while some T cell responses mounted during chronic infection by MHC-matched MCM are similar, the majority of responses are highly variable. Shared responses detected in this study between MHC-matched MCM were directed against epitopes that had previously elicited relatively dominant responses in MCM with the same MHC class I haplotype, suggesting that the factors that influence dominance may influence the reproducibility of responses as well. This may be an important consideration for future T cell-based vaccines aiming to consistently and reproducibly elicit protective T cell responses.

## Findings

Genome-wide association studies have implicated the major histocompatibility complex (MHC) class I genes, and by extension the CD8+ T cell responses they restrict, in modulating durable control of human immunodeficiency virus (HIV) [1]. The role of CD8+ T cells in viral control has been confirmed in nonhuman primates using simian immunodeficiency virus (SIV) [2,3]. Despite the correlation between CD8+ T cell responses and HIV/SIV control, a clear understanding of what constitutes an effective T cell response against these viruses remains elusive [4]. This is further confounded by complex host MHC genetics and interhost viral variability, which complicate the process of differentiating between the influence of genetic and stochastic factors in immune responses and disease progression [5].

Researchers have studied HIV-infected MHC-identical siblings and monozygotic twins to assess how genetic and stochastic effects influence HIV-specific immune responses, finding that T cell responses mounted in acute infection are strikingly similar, but diverge later in MHC-identical individuals [6-8]. This divergence may be attributed to stochastic expansions of HIV-specific CD8+ T cells with specific T cell receptors [9]. Opportunities to study HIV in this context, however, are rare and limited to single pairs of individuals, preventing further insights into immune responses and viral evolution in identical host MHC backgrounds.

SIV infection of non-human primates is a well-established model of HIV infection, allowing researchers to infect macaques with clonal, pathogenic virus and study SIV-specific T cell responses in animals with particular MHC class I alleles [10]. Mauritian cynomolgus macaques (MCM) are unique because of their limited MHC genetic diversity when compared to humans and other macaque populations. The MHC genetics of MCM have been well defined and most of the MHC class I diversity can be accounted for by only seven haplotypes, termed M1-M7 [11-13]. Similarly, the MHC class II diversity of MCM is very limited [14]. This allows for MCM to be matched for both their MHC class I and class II haplotype and provides the rare opportunity to study both CD4+ and CD8+ host T cell responses, against clonal SIV in cohorts of MHC-matched individuals.

In this study, we used 822 overlapping 15-mers spanning the entire SIVmac239 proteome to measure T cell responses by gamma interferon (IFN-γ) enzyme-linked immunospot (ELISPOT) in groups of MHC class I and class II-matched animals. We hypothesized that animals who shared identical MHC genes would mount T cell responses with similar peptide specificity and magnitude at matching time-points, i.e. that responses in MHC-matched animals are "reproducible". Our use of MCM allowed us to closely compare chronic T cell response reproducibility among genetically similar animals.

**Figure 1:**
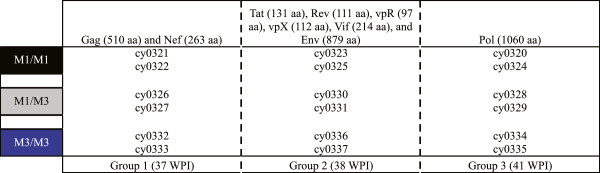
**MHC-matched animals pairs.** A total of 18 MCM evenly split between the M1/M1, M1/M3, and M3/M3 haplotypes were screened using an individual peptide IFN-γ ELISPOT assay between 37 and 41 WPI. Animals were divided into 3 groups of 6 MCM and screened against a third of the SIVmac239 proteome. In each group, 2 MCM of each haplotype were represented.

We used 18 MCM homozygous or heterozygous for the M1 or M3 MHC class I haplotype (6 M1/M1, 6 M1/M3, and 6 M3/M3) in an individual 15-mer peptide IFN-γ ELISPOT assay. Animals matched for their MHC class I haplotype also were matched for their class II haplotype. Animals were split into three groups, each containing an M1/M1 pair, an M1/M3 pair, and an M3/M3 pair, for a total of six animals per group. Each group was screened for responses against one third of the SIVmac239 proteome. Thus, each peptide was tested in three pairs of animals between 37 and 41 weeks post-infection (WPI) (Figure 1). ELISPOT assays were performed as previously described [12,15,16] and positive responses were defined as being greater than 75 spot-forming cells (SFC) per million peripheral blood mononuclear cells (PBMC) above background. One M1/M1 MCM pair, cy0321 and cy0322, screened against the Gag and Nef proteins was excluded from comparisons due to high background IFN-γ secretion in cy0322, over 100 SFCs, averaging 360 SFCs in the negative control wells. The average background across the entire group was 24.3 SFCs with a range from 0 to 361 SFCs; however, when we excluded cy0322, the average background was 5.2 SFCs and ranged from 0 to 16 SFCs. No responses were detected in the three MCM pairs screened against the Pol protein (data not shown); thus, comparisons could not be made within these pairs.

**Figure 2:**
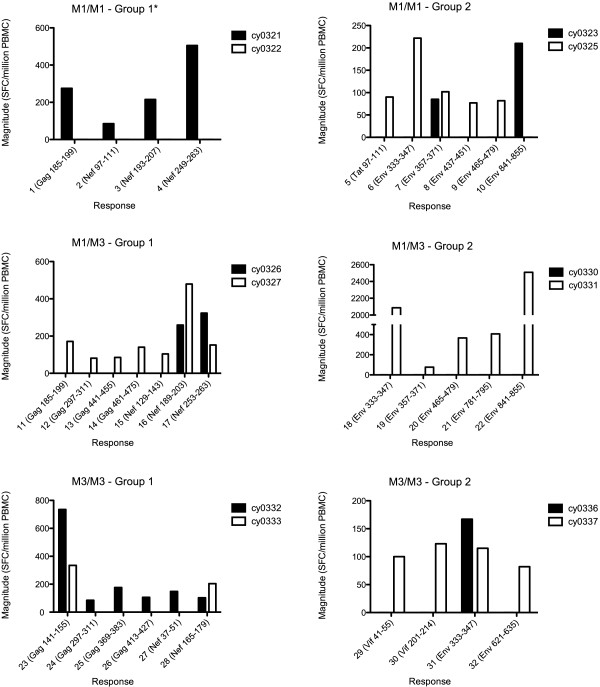
**SIVmac239-specific T cell responses detected by individual IFN- γ ELISPOT assays.** Comparison of the location and magnitude of SIVmac239-specific T cell responses detected by individual peptide IFN-γ ELISPOT assays from 18 MCM with the M1/M1, M1/M3, or M3/M3 haplotype. Animals were divided into pairs by MHC class I haplotype and each pair was screened against approximately one third of the SIV proteome. Responses against the most immunogenic peptides within amino acid regions that elicited a response are shown and responses are numbered for reference in Figure 2. Because no responses were detected by ELISPOT in group 3 animals screened against the Pol protein, these animals are not shown. Due to high background IFN-γ secretion by cy0322, the M1/M1 Group 1 pair of cy0321 and cy0322 (asterisk) was not included in our comparison of responses detected by ELISPOT; however, responses mounted by cy0321 are shown.

Surprisingly, ELISPOT assays revealed a lack of concordance in IFN-γ-producing T cell responses between MHC-matched individuals screened against the same proteins. None of the 5 MHC-matched pairs that could be compared exhibited identical T cell specificity or magnitude against SIVmac239 (Figure 2). Indeed, the M1/M1 pair shared only 1 of 6 distinct responses (#7 in Figure 2), while M1/M3 pairs shared 2 of 12 distinct responses (#16 and #17 in Figure 2), and M3/M3 pairs shared 3 of 10 distinct responses (#23, #28, and #31 in Figure 2). Three of six shared responses were directed against previously defined epitopes: Nef _196–203_ HW8, Gag _146–154_ HL9, and Env _338–346_ RF9 [5,11]. The remaining three shared responses were directed against Env 357–371, Nef 253–263, and Nef 165–179 (Figure 2). No correlation in response magnitude between animals was observed for shared responses (R^2^ = 0.197, P = 0.3782).

**Figure 3:**
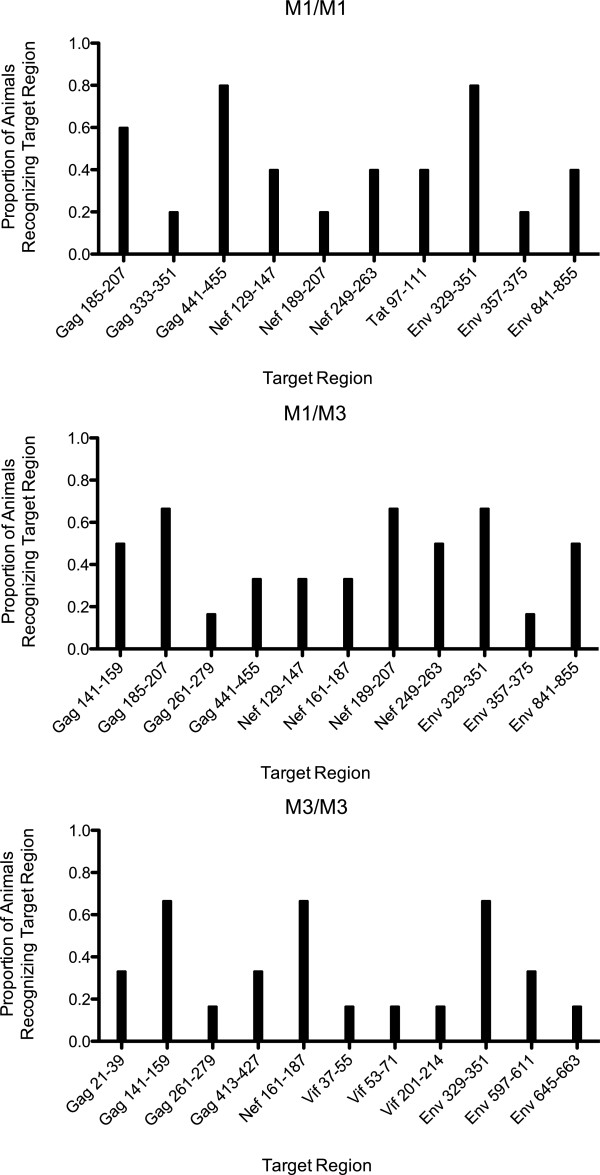
**52 WPI SIVmac239-specific T cell response reproducibility in MHC-matched MCM.** On the x-axis are regions targeted by IFN-γ-secreting T cells in at least one animal of that haplotype, as measured by 52 WPI pooled peptide matrix ELISPOT. On the y-axis is the proportion of animals within that MHC-matched group that mounted a response to each region. In each group, six animals were compared, with the exception of the M1/M1 group. Due to high background IFN-γ-secretion, cy0322 was excluded from comparison with other M1/M1 animals.

We next expanded our analysis, examining previously collected 52 WPI full proteome matrix ELISPOT data from this cohort [5]. This allowed for the comparison of chronic T cell response reproducibility in larger groups of MHC-matched MCM and across the full SIVmac239 proteome. Again, high non-specific IFN-γ secretion in cy0322 prohibited detection of positive responses in this animal. Animals were grouped by MHC haplotype and for each region targeted by at least one animal, the proportion of MHC-matched animals mounting a response to that same region was calculated (Figure 3). None of the responses mounted in any of the three MHC-matched groups were detected in all of the animals screened, and of the 32 regions targeted by T cells from at least one animal at 52 WPI, 20 were recognized by two or fewer MHC-matched MCM (Figure 3). This further supports our earlier observation of chronic-phase T cell response discordance between MHC-matched MCM. Moreover, these results were confirmed in the M3/M3 animals that had similar disease progression and viral loads, thus, eliminating potential confounding effects of some animals controlling viral replication.

The availability of chronic-phase ELISPOT data from both individual peptide ELISPOT assays and pooled peptide matrix ELISPOT assays in the same cohort of animals provides an opportunity to assess the concordance and sensitivity of these two assays. For each animal, only the responses mounted within the proteins screened in the individual peptide assays were compared. Of the 38 responses detected in individual peptide ELISPOT, 21 (55.3%) were again detected in the same animal at 52 WPI via matrix ELISPOT. Nine responses, however, were detected by matrix ELISPOT that were not observed in our individual peptide ELISPOT assays. This suggests that the variability in responses detected may be a product of differences in sampling time point, rather than a difference in assay sensitivity.

The discordance in chronic T cell responses made by MHC-matched MCM was an intriguing finding, and one that we explored further. Full proteome matrix ELISPOT data from this cohort has allowed for the relative dominance of each response detected in our individual peptide ELISPOT assays to be examined. Here, we defined dominant responses as constituting 25 percent or more of the overall magnitude of the SIV-specific T cell response in an animal at 24 or 52 WPI, as detected by matrix ELISPOT. Interestingly, all six of the shared responses identified by individual peptide ELISPOT were dominant in at least one animal of the same haplotype, while only nine of the 22 non-shared responses were dominant in at least one animal of the same haplotype (Additional File 1: Table S1).

**Figure 4:**
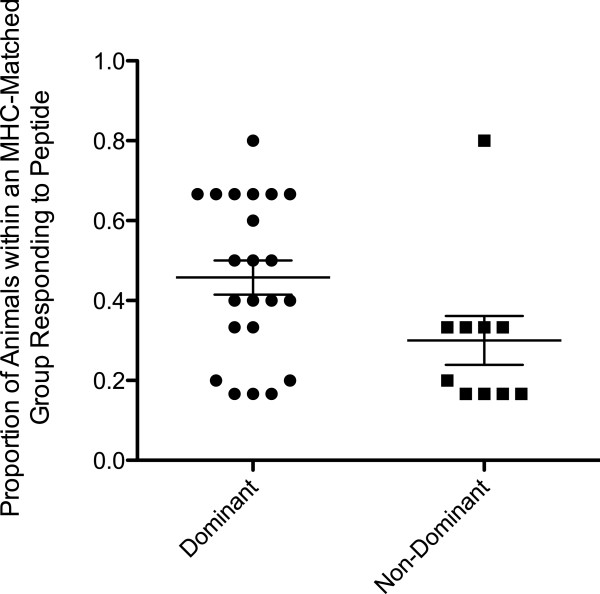
**Dominant and Non-dominant T Cell Response Reproducibility.** Of the 32 peptides that elicited a T cell response at 52 WPI within a given MHC-matched group, 22 induced a response that was dominant in at least one animal within the same MHC class I haplotype. The proportion of animals mounting a response against these peptides was significantly greater than proportion of animals within an MHC-matched group that mounted a response against a peptide that did not elicit a dominant response in any animals with that class I haplotype (P < 0.05). Responses were detected by full proteome pooled peptide matrix ELISPOT at 52 WPI. Lines represent the mean proportion of animals within an MHC-matched group responding to target region and error bars represent the standard error of the mean.

When comparing more animals at 52 WPI across the entire SIVmac239 proteome, we saw a similar pattern of T cell response reproducibility. Peptides that elicited a dominant response in at least one animal within an MHC-matched group were significantly more likely to elicit a T cell response in the other animals of that group than peptides that did not elicit a dominant response in any animals within an MHC-matched group (P < 0.05, Figure 4). This finding further supports earlier observations derived from individual peptide ELISPOT assays and suggests that dominant responses may be more reproducible in chronic-phase SIVmac239 infection of MCM.

This study is, to our knowledge, the first to use MHC-matched animals to screen all known SIVmac239 proteins using only individual 15-mer peptides and to closely compare the chronic-phase T cell responses mounted by MHC-matched animals. ELISPOT revealed that, while some responses from MHC-matched animals were shared, the majority of IFN-γ secreting T cell responses were not consistently detected between animals during chronic SIV infection, and the magnitude of shared responses varied considerably. These findings are consistent with research on HIV-infected monozygotic twins and MHC-matched siblings [6-8].

Dominant responses detected by ELISPOT were the exception to this variability. This is consistent with the highly reproducible immunodominant T cell response hierarchies of inbred mice to pathogens such as influenza A, lymphocytic choriomeningitis virus, and vaccinia virus, among others [17]; and to a lesser extent, immunodominant HIV-specific response reproducibility in humans [17-19]. Given the findings of our study, it is plausible that factors such as antigen processing and presentation, antigen-MHC class I binding affinity, T cell avidity for the MHC class I-peptide complex, and naïve T cell precursor frequency which contribute to the immunodominance of certain CD8+ T cell responses [20] may also contribute strongly to their reproducibility in MHC-matched MCM. This may provide insight into ways in which responses against subdominant epitopes, which have been implicated in the control of HIV/SIV [2,21], may be more reliably elicited. Despite the variability of subdominant responses in MCM, it has been shown in other models that immunodominance hierarchies can be altered to favor effective responses against subdominant epitopes [22] and further research in this area may inform the design of HIV vaccines capable of reliably inducing a variety of specific protective T cell responses in MHC-diverse populations.

## Abbreviations

MHC: Major histocompatability complex; HIV: Human immunodeficiency virus; HIV-1: Human immunodeficiency virus type 1; SIV: Simian immunodeficiency virus; MCM: Mauritian cynomolgus macaque; IFN-γ: Gamma interferon; ELISPOT: Enzyme-linked immunospot; WPI: Weeks post-infection; SFC: Spot-forming cells; PBMC: Peripheral blood mononuclear cells.

## Competing interest

There are no competing interests to report for this manuscript.

## Authors’ contributions

BC and NP collected the data and drafted the manuscript. MB and JG participated in the design of the study, contributed to the immunoassays and data collection, and were involved in the revision of the manuscript. JW and EC participated in the immunoassays. TF and DO conceived of the study and participated in its design and coordination. All authors read and approved of the final manuscript.

## Supplementary Material

Additional file 1: Table S1 Regions of the SIVmac239 proteome that elicited T cell responses. Regions of the SIVmac239 proteome that elicited T cell responses in our cohort and the individual peptide within each region that induced the strongest response. Responses were detected via individual peptide IFN-γ ELISPOT. The magnitude of the response against the most immunogenic peptide is shown and responses are numbered for reference in Figure 1 and Figure 2. Responses in bold italics are ones that have been defined as dominant. Click here for file
