# Supplementary material for: T cell response specificity and magnitude against SIVmac239 are not concordant in major histocompatibility complex-matched animals
Source: Retrovirology. 2013 Oct 24;10:116. doi: 10.1186/1742-4690-10-116 (PMC3874790; doi:10.1186/1742-4690-10-116)
Supplement: Additional file 1: Table S1 — Regions of the SIVmac239 proteome that elicited T cell responses. Regions of the SIVmac239 proteome that elicited T cell responses in our cohort and the individual peptide within each region that induced the strongest response. Responses were detected via individual peptide IFN-γ ELISPOT. The magnitude of the response against the most immunogenic peptide is shown and responses are numbered for reference in Figure 1 and Figure 2. Responses in bold italics are ones that have been defined as dominant. [file 1742-4690-10-116-S1.pdf]

Supplemental Table 1.

Response Number	Response	Animal	Haplotype	Most Immunogenic peptide	Magnitude of Response (SFC/million PBMC)
1	***Gag 185-199***	***cy0321***	***M1/M1***	***Gag 185-199***	***275***
2	Nef 97-115	cy0321	M1/M1	Nef 97-111	85
3	***Nef 189-207***	***cy0321***	***M1/M1***	***Nef 193-207***	***215***
4	***Nef 249-267***	***cy0321***	***M1/M1***	***Nef 249-263***	***505***
5	***Tat 97-111***	***cy0325***	***M1/M1***	***Tat 97-111***	***90***
6	***Env 329-351***	***cy0325***	***M1/M1***	***Env 333-347***	***222***
7	***Env 357-371***	***cy0323***	***M1/M1***	***Env 357-371***	***85***
7	***Env 357-375***	***cy0325***	***M1/M1***	***Env 357-371***	***102***
8	Env 437-455	cy0325	M1/M1	Env 437-451	77
9	Env 465-479	cy0325	M1/M1	Env 465-479	82
10	***Env 841-855***	***cy0323***	***M1/M1***	***Env 841-855***	***210***
11	***Gag 185-199***	***cy0327***	***M1/M3***	***Gag 185-199***	***171***
12	Gag 297-315	cy0327	M1/M3	Gag 297-311	81
13	***Gag 441-455***	***cy0327***	***M1/M3***	***Gag 441-455***	***85***
14	Gag 461-475	cy0327	M1/M3	Gag 461-475	140
15	***Nef 129-147***	***cy0327***	***M1/M3***	***Nef 129-143***	***104***
16	***Nef 189-207***	***cy0326***	***M1/M3***	***Nef 189-203***	***259***
16	***Nef 189-207***	***cy0327***	***M1/M3***	***Nef 189-203***	***479***
17	***Nef 249-263***	***cy0326***	***M1/M3***	***Nef 253-263***	***323***
17	***Nef 249-263***	***cy0327***	***M1/M3***	***Nef 253-263***	***152***
18	***Env 329-351***	***cy0331***	***M1/M3***	***Env 333-347***	***2087***
19	Env 357-375	cy0331	M1/M3	Env 357-371	77
20	Env 465-479	cy0331	M1/M3	Env 465-479	367
21	Env 781-795	cy0331	M1/M3	Env 781-795	407
22	***Env 841-855***	***cy0331***	***M1/M3***	***Env 841-855***	***2510***
23	***Gag 141-159***	***cy0332***	***M3/M3***	***Gag 141-155***	***735***
23	***Gag 141-159***	***cy0333***	***M3/M3***	***Gag 141-155***	***335***
24	Gag 297-315	cy0332	M3/M3	Gag 297-311	85
25	Gag 369-383	cy0332	M3/M3	Gag 369-383	176
26	Gag 413-427	cy0332	M3/M3	Gag 413-427	106
27	Nef 25-51	cy0332	M3/M3	Nef 37-51	148
28	***Nef 165-187***	***cy0332***	***M3/M3***	***Nef 165-179***	***103***
28	***Nef 161-183***	***cy0333***	***M3/M3***	***Nef 165-179***	***204***
29	Vif 37-55	cy0337	M3/M3	Vif 41-55	100
30	***Vif 201-214***	***cy0337***	***M3/M3***	***Vif 201-214***	***123***
31	***Env 329-351***	***cy0336***	***M3/M3***	***Env 333-347***	***167***
31	***Env 329-351***	***cy0337***	***M3/M3***	***Env 333-347***	***115***
32	Env 621-639	cy0337	M3/M3	Env 621-635	82
